# Capillary-Seeding Crystallization and Preliminary Crystallographic Analysis of a Solvent-Tolerant Elastase from *Pseudomonas aeruginosa* Strain K

**DOI:** 10.3390/ijms140917608

**Published:** 2013-08-28

**Authors:** Mohd Shukuri Mohamad Ali, Zatty Syamimi @ Adura Mat Said, Raja Noor Zaliha Raja Abd Rahman, Adam Leow Thean Chor, Mahiran Basri, Abu Bakar Salleh

**Affiliations:** 1 Enzyme and Microbial Technology Research Centre, Faculty of Biotechnology and Biomolecular Sciences, Universiti Putra Malaysia, Serdang UPM 43400, Selangor, Malaysia; E-Mails: zattyadura@gmail.com (Z.S.A.M.S.); rnzaliha@biotech.upm.edu.my (R.N.Z.R.A.R.); adamleow@biotech.upm.edu.my (A.L.T.C.); mahiran@science.upm.edu.my (M.B.); abubakar@biotech.upm.edu.my (A.B.S.); 2 Department of Biochemistry, Faculty of Biotechnology and Biomolecular Sciences, Universiti Putra Malaysia, Serdang UPM 43400, Selangor, Malaysia; 3 Department of Microbiology, Faculty of Biotechnology and Biomolecular Sciences, Universiti Putra Malaysia, Serdang UPM 43400, Selangor, Malaysia; 4 Department of Cell & Molecular Biology, Faculty of Biotechnology and Biomolecular Sciences, Universiti Putra Malaysia, Serdang UPM 43400, Selangor, Malaysia; 5 Faculty of Sciences, Universiti Putra Malaysia, Serdang 43400, Selangor, Malaysia

**Keywords:** elastase strain K, organic solvent tolerant, seeding technique, capillary counter diffusion

## Abstract

Seeding is a versatile method for optimizing crystal growth. Coupling this technique with capillary counter diffusion crystallization enhances the size and diffraction quality of the crystals. In this article, crystals for organic solvent-tolerant recombinant elastase strain K were successfully produced through microseeding with capillary counter-diffusion crystallization. This technique improved the nucleation success rate with a low protein concentration (3.00 mg/mL). The crystal was grown in 1 M ammonium phosphate monobasic and 0.1 M sodium citrate tribasic dihydrate pH 5.6. The optimized crystal size was 1 × 0.1 × 0.05 mm^3^. Elastase strain K successfully diffracted up to 1.39 Å at SPring-8, Japan, using synchrotron radiation for preliminary data diffraction analysis. The space group was determined to be monoclinic space group P12_1_1 with unit cell parameters of *a* = 38.99 Ǻ, *b* = 90.173 Å and *c* = 40.60 Å.

## 1. Introduction

Crystallographers have developed many protein crystallization methods. More than 20 techniques have been reported; the most commonly used techniques are microbatch, counter diffusion, dialysis and hanging and sitting drop [1–4]. The method developed from the crystallization phase diagram is the seeding method [5,6], in which authors attempted to decouple nucleation and growth. Another crystallization method, the counter-diffusion method, provides better crystallization conditions for growing crystals [7]. These methods were developed for enhancing the quality and size of the crystals for atomic structure determination.

Obtaining a high-quality crystal that is capable of producing high resolution diffraction is critical to crystallography [5]. Interpreting the strong diffraction data recorded from large protein crystals provides detailed information on the atomic structure and specific molecular interactions. However, determining suitable crystallization conditions for a particular protein remains a highly empirical process. Crystallization procedures have been investigated particularly for better protein crystal morphology and internal packing as well as to influence crystal growth. Studies on the fundamental biochemical and molecular biology of a protein at the molecular level may support future research in developing valuable products for people.

Thus, the organic solvent-tolerant elastase strain K was crystallized. Organic solvent-tolerant proteases from the bacterium *Pseudomonas aeruginosa* strain K were previously isolated and screened in our laboratory by Geok *et al.* [8]; Rahman *et al.* [9] further purified and characterized this protease. The purification results show that this bacterium produces three protease types. One of these proteases was highly similar to the elastase from *P. aeruginosa.* Exploring the stability of this enzyme (elastase from *P. aeruginosa* strain K) and using molecular genetics for gene overexpression were well documented by Wong *et al.* [10]. Further investigations to characterize the purified recombinant elastase strain K showed its capability for activity in hydrophilic organic solvent media [11]. Given the previous investigations, our research will focus on recombinant elastase strain K crystallization and structure determination to discern its structural features as an organic solvent-stable enzyme. In this report, we present the crystallization technique used to produce high-diffraction quality crystals for elastase strain K and preliminary X-ray crystallographic data.

## 2. Results and Discussion

### 2.1. Preparing Purified Enzyme Elastase Strain K

**Figure 1:**
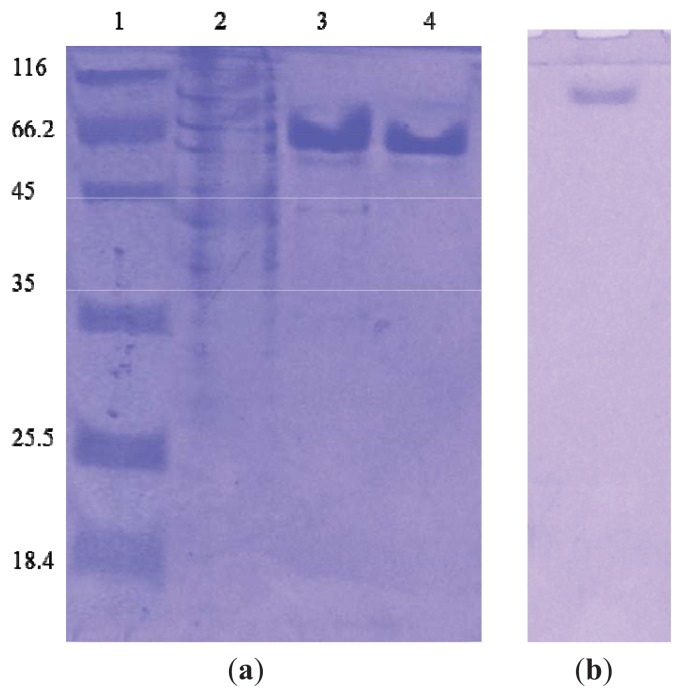
SDS-PAGE profile for purified elastase after series of purification steps. (**a**) Lane 1: protein molecular weight marker in kDa [Unstained Protein Molecular Weight Marker (Fermentas, Glen Burnie, MD, USA)]; Lane 2: crude sample; Lane 3: hydrophobic interaction chromatography fraction (HIC); and Lane 4: ion exchange chromatography (IEC) fraction. (**b**)The homogeneity was confirmed via native-PAGE.

Elastase strain K was purified through a series of steps conducted in a cold room to avoid autolysis. Purification efficiency was analyzed through SDS-PAGE. The protein was analyzed at various steps from the crude sample (Figure 1a; lane 2) to the purified enzyme (Figure 1a; lanes 3 and 4). Hydrophobic interaction chromatography (HIC) and ion exchange chromatography (IEC) were clearly highly efficient as demonstrated using gel analysis, which depicts bulk removal of a contaminant (unwanted band) after such purification steps. IEC was the second purification step used to further refine the protein sample to final purity. In addition, the purity of recombinant elastase strain K was also examined using native-PAGE (Figure 1b), where the protein migrated as a single band. The highest protein concentration generated was 3 mg/mL, as determined using the Bradford assay.

### 2.2. Three-Dimensional Crystallization and Analysis

Elastase strain K crystals were screened using various crystallization kits and the vapor diffusion method. The crystals were grown at 20 °C. Elastase crystals were generated using different crystallization conditions. Small crystals appeared under different crystallization conditions on the ninth day of incubation (Table 1). Based on the successful crystallization conditions, the salt component in the precipitant agent was observed for each condition. These anti-chaotropic salts dehydrate proteins using the “salting-out” principle [12,13]. Concentrated sulfate and phosphate ions in solution yield sufficient charges to “dehydrate” the elastase solution. Based on the Hofmeister series, SO_4_^2−^ and HPO_4_^2−^ are the two most effective ions for salting out and these ions are most commonly used for precipitation [14]. Furthermore, an ion concentration ranging from 0.8 to 1.2 M is sufficient to “strip-off” the water bound to elastase and to decrease its water solubility.

### 2.3. Enhancing the Elastase Crystal Size through Seeding

**Figure 2:**
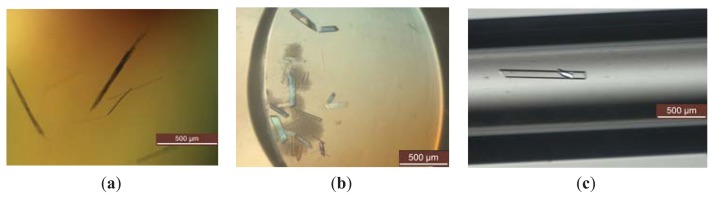
Enhanced elastase crystal size. Elastase crystal. (**a**) Crystal growth during crystal screening; (**b**) crystal growth using the hanging drop method; and (**c**) crystals in the capillary using the microseeding counter-diffusion method.

**Figure 3:**
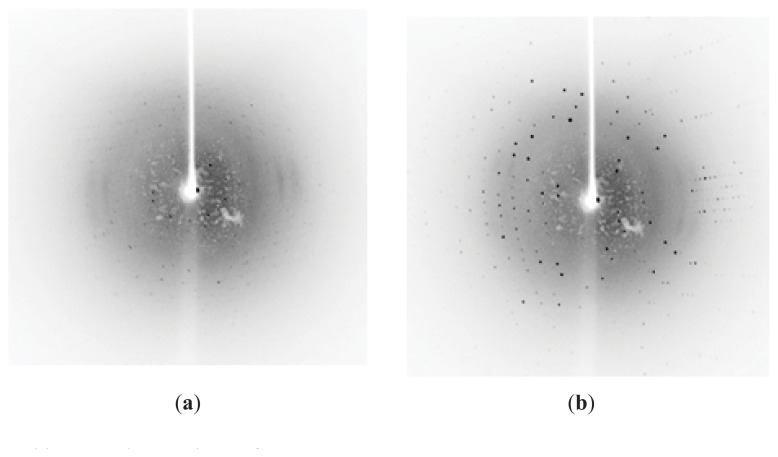
Comparison of the elastase strain K diffraction patterns. (**a**) Weak diffraction data were generated due to poor crystal quality; thus, no findings were derived; (**b**) The diffraction spots markedly improved for crystals grown using the seeding-counter diffusion methods.

Tiny, twinned and sometimes clustered needlelike crystals during the crystal screening did not yield promising diffraction-quality data. Occasionally, the identical protein droplets that typically produce crystals are inconsistent. Therefore, crystal growth is optimized by the microseeding technique. The seeding method is the most systematic approach for optimizing crystallization conditions. Bergfors [5] stated that seeding is a worthwhile optimization step at early stages and that its application an improve crystal quality. Theoretical and empirical investigations were published by Stewart *et al.* [15], who described successful protein crystallization through seeding to generate better diffracting crystals. During initial crystallization condition screening, elastase crystals with promising quality, as determined through simple scan X-ray diffraction, were generated in 0.1 M tribasic sodium citrate dihydrate pH 5.6 and 1.0 M monobasic ammonium phosphate (Figures 2a and 3a). These crystals were selected for pulverization to generate a seed and induce nucleation in protein droplets. The seeds were transferred using a probe (human hair) and streaking the seed into a new identical reservoir that previously formed a crystal. After day one, several crystals were observed for each identical condition with improved crystal morphology (Figure 2b). Single, rectangular crystals were grown to approximately 0.2 × 0.1 × 0.01 mm^3^. The cluster crystal problem was solved by growing the crystals as a single crystal due to introducing a diluted seed stock that can control the levels of nuclei drawn into a droplet [5]; thus, the nuclei can grow freely without additional overlapping nuclei. Moreover, this technique also indirectly eliminates self-nucleation in the droplet; hence, the crystal can grow into a larger crystal without competition from new protein nuclei. By applying ready-nuclei in the droplet, the crystal formation success rate will increase even at low elastase protein concentrations.

### 2.4. Favorable Crystal Growth Conditions

However, further protein crystal optimization was used to refine the crystal quality for X-ray diffraction and structural determination at atomic resolution [4]; crystallization is a complex and multiparametric process. Biertumpfel *et al.* [16] and Garcia-Ruiz [7] stated that counter diffusion is an efficient technique for producing many high-quality macromolecular crystals. For typical crystallization techniques, convection disturbs the concentration gradient and mass transport. By applying this method convective mixing was avoided thus allowing mass transport of the protein and precipitant agent molecules for interaction through diffusion [17]. This technique is efficient because it facilities suitable kinetic parameters [18]. As the solutions interact, they will act in accordance with the protein’s solubility dependence on the precipitating agent concentration and form a non-homogenous spatial distribution for crystals as the interaction continues. However, the elastase strain K concentration was too low for the capillary counter diffusion method. For counter diffusion, the protein concentration in the capillary must be between 4 to 20 mg/mL or higher [17]. Thus, gradual supersaturation can be generated continuously through diffusion, which facilities protein nucleation and crystal growth.

Introducing a seeding technique with counter-diffusion crystallization in a capillary was successful for enhancing protein crystal quality though both techniques, which comprise distinct approaches that can operate independently. The elastase concentration herein was insufficient to overcome the nucleation energy barrier and form crystals because capillary counter-diffusion requires highly concentrated protein. However, this problem was overcome through microseeding, which increases the chance of crystal hits. Seeding with counter diffusion was successful in a capillary because the seeds were stable during mixing at the protein concentration in the capillary. Microseeding enhances nucleation when high protein concentrations are unavailable [19]. The crystals grew inside the capillary to 1 × 0.1 × 0.05 mm^3^ using 1 M ammonium phosphate monobasic and 0.1 M sodium citrate tribasic dihydrate pH5.6 as the precipitant. After one week of incubation, this crystal improved in size and diffraction, as shown in Figures 2c and 3b respectively.

### 2.5. Preliminary X-ray Diffraction

The diffraction data show that the elastase strain K crystals adopt the monoclinic space group P12_1_1 with unit cell parameters of *a* = 38.99 Å, *b* = 90.173 Å, *c* = 40.60 Ǻ and β = 113.81°. The data were 99.9% complete at a 1.39 Å resolution. Solvent analysis via the Matthews coefficient [20] estimated that approximately 36.15% corresponds to one monomer per asymmetric unit. The structure for the organic solvent-tolerant elastase strain K will be elucidated using molecular replacement and the computational CCP4 program package [21] with the *Pseudomonas aeruginosa* elastase crystal structure (1EZM) [22] as the template. The diffraction data quality for elastase strain K improved more compared with previous work by Thayer [22]. However, previously reported elastase have not been organic-solvent tolerant. The data collection statistics was as shown in Table 2.

## 3. Experimental Section

### 3.1. Elastase Strain K Serial Purification

The recombinant elastase strain K was overexpressed in *E. coli* KRX/pCon2(3) and purified to homogeneity as described by Rahman *et al.* [11] with slight modifications. Three liters of *E. coli* KRX/pCon2(3) culture was harvested through centrifugation and the precipitated cells were dissolved in reaction buffer (50 mM Tris-Cl, pH 8.5) before sonication to release the enzyme. The lysed culture was centrifuged at 15,000× *g* for 10 min at 4 °C. The soluble enzyme was diluted by adding reaction buffer with ammonium sulfate and EDTA, which generated a final protein solution with 50 mM Tris-Cl (pH 8.5), 1 M (NH_4_)_2_SO_4_, and 10 mM EDTA.

The crude enzyme was loaded on Butyl-S sepharose fast flow resin (GE Healthcare, Uppsala, Sweden) packed in an XK16 column (GE Healthcare, Uppsala, Sweden) that was pre-equilibrated with 50 mM Tris-Cl (pH 8.5) and 1 M (NH_4_)_2_SO_4_. Seven column volumes (CV) of this buffer were used to wash the unbound protein from the column. Next, the target protein (bound protein) was eluted through salt gradient elution from high to low ionic strength using (NH_4_)_2_SO_4_ from 1 to 0 M. The eluted fractions with the protein were pooled and the buffer exchanged (50 mM Tris-Cl, pH 9) using Sephadex G-25 resin (GE Healthcare, Uppsala, Sweden). For further refinement, ion exchange chromatography (IEC) was employed using Q-Sepharose fast flow resin (GE Healthcare, Uppsala, Sweden), which was equilibrated with 50 mM Tris-Cl, pH 9 at a 0.5 mL/min flow rate. The protein eluted from the column using linear gradient elution with NaCl from 0 to 0.5 M. Consequently, the protein fractions were pooled and dialyzed against 50 mM Tris-Cl, pH 8.7 at 4 °C to remove the salt. Prior to crystallization, the purified elastasestrain K was concentrated using a Vivaspin 20 centrifugal concentrator (Sartorius Stedim Biotech GmbH, Gottingen, Germany) to approximately 3 mg/mL. The final protein concentration was measured using a Bradford assay kit (Amresco, Solon, OH, USA) and the purity was analyzed through 12% SDS-PAGE [23].The concentrated protein was aliquoted into sterile test tubes in 50–100 μL fractions and stored at −20 °C. Prior to the crystallization experiments, the protein aliquots were thawed on ice and centrifuged to remove aggregated protein.

### 3.2. Crystallization Trials

A sparse matrix approach (Crystal screen and Crystal screen 2 reagent kits), random sparse matrices (Emerald BioSystems Wizard™ Screens I and II) and Molecular Dimensions crystallization kits were used for the initial protein crystallization trials.

Crystallization screens were performed via the sitting drop and hanging drop vapor diffusion methods. For both methods, 6 μL (1:1 ratio of protein and crystallization solution) was equilibrated against the crystallization solutions at 20 °C. The crystallization plates were examined every day to discern changes in the droplet.

### 3.3. Crystallization Method

#### 3.3.1. Preparing the Seed Stock for Crystallization

A crystal grown using the vapor diffusion method was used as the seed crystal to enhance the reproducibility of crystal production. The seed stock was prepared in accordance with Luft and DeTitta [24] with slight modifications. The seed stock was prepared by crushing the crystal using a probe in the well plate for vapor diffusion under a stereomicroscope, and then it was transferred into an autoclaved Eppendorf tube. Another 5 μL of the crystallization solution was pipetted into the droplet and transferred to the same Eppendorf tube. This step was repeated twice to ensure that the crushed crystal was fully transferred into the tube. The crushed crystals were pulverized by adding a glass bead to the Eppendorf tube and vortexing for approximately 2 min to ensure that each crystal was fully ground. This experiment was conducted on ice because the seed was unstable. The seed stocks were stored at −80 °C.

Prior to seeding, the seed crystal was thawed on ice and vortexed. Seeding was performed to enhance crystal growth. Streak-seeding was employed using human hair as a crystal wand to transfer the seeds into new drops. The protein droplet and reservoir used herein were prepared as previously indicated for the crystallization trial. A seeding wand was used to touch the seed stocks and dislodge the crystal nuclei into the protein droplet. Next, the plate was sealed with tape and incubated at 20 °C. The crystals grown in the droplet were validated using Izit crystal dye (Hampton Research, Aliso Viejo, CA, USA).

#### 3.3.2. Seeding-Counter Diffusion Crystallization

To generate ideal conditions for crystal optimization, another method was employed that couples seeding with capillary counter diffusion using a Crystal-Tube Kit (Confocal Science Inc., Tokyo, Japan). Inspired by Gavira *et al.* [19], 10 μL of protein was filled in 0.5 mm diameter capillaries, which were dipped into 2 μL of a seeding droplet. Next, the capillary was sealed at one end with clay and another end was attached to anagarose-gel tube. The capillary was placed into an aspirating tube with crystallization solution by dipping the agarose gel end into the crystallization solution. The crystallization apparatus was then incubated at 20 °C and was observed daily for crystal growth.

#### 3.3.3. Harvesting Protein Crystals in the Capillary

Harvesting the elastase crystal was performed in accordance with the manufacturer’s instructions, (Crystal-Tube, Confocal Science Inc., Tokyo, Japan). The capillary crystal-tube with the elastase crystal was cut with a razor blade 1 to 2 mm away from the target crystal. Next, the capillary was gently rinsed with mother liquor, such that the crystal was slowly removed from the capillary. The crystal was placed in cryoprotectant (30% glycerol in mother liquor).

### 3.4. X-ray Data Collection and Processing

The preliminary X-ray diffraction was tested using an in-house Bruker X8 PROTEUM biological single crystal 103 diffractometer system with a MICROSTAR microfocus rotating anode generator 104 (Bruker, Bremen, Germany). Prior to X-ray diffraction, the elastase strain K crystal was mounted using cryoloops (0.3 mm loop diameter; Hampton Research, Aliso Viejo, CA, USA) in cryoprotectant (30% glycerol in mother liquor) and flash cooled in a 100 K nitrogen stream.

For data collection, elastase strain K X-ray diffraction data were collected via synchrotron radiation at beamline BL44XU, SPring-8 (Sayo, Hyogo, Japan). A total of 360 images with 1.0° oscillation were collected with a one second per image exposure time and a crystal-to-detector distance of 146.80 mm. The data were indexed, integrated and scaled using HKL-2000 [25].

## 4. Conclusions

In conclusion, the organic solvent-tolerant elastase strain K was successfully crystallized and diffracted. Crystal optimization techniques were used to improve the quality of the elastase crystals. The good-quality data must be solved to understand the protein-solvent interaction. The structure may be useful for developing a better enzyme suitable for academic and industrial applications in the presence of organic solvent. Elucidating the elastase strain K structure is in progress.

## Figures and Tables

**Table 1:** The lists of the crystallization screening conditions that produced crystals for elastase strain K at 20 °C. The crystals generated using the listed conditions were small, and diffraction spots were only slightly generated.

Crystal screen	Condition number	Chemical composition
Wizard		
I	27	1.2 M sodium phosphate/0.8 M potassium phosphate 100 mM CAPS pH 10.5 0.2 M Lithium sulfate
II	9	2 M ammonium sulfate 100 mM Phosphate citrate pH 4.2
II	19	1.6 M Sodium phosphate/0.4 M potassium phosphate 100 mM Phosphate citrate pH 7.5

Molecular dimension		
II	22	1 M ammonium sulfate 0.1 M Tris pH 8
II	23	1.5 M ammonium sulfate 0.1 M sodium acetate pH 5

Hampton Research		
I	3	0.4 M ammonium phosphate monobasic
I	11	0.1 M sodium citrate tribasic dehydrate pH 5.6 1 M ammonium phosphate monobasic
I	35	0.1 M HEPES sodium pH 7.5 0.8 M Potassium phosphate monobasic/0.8 M sodium phosphate monobasic monohydrate
I	39	0.1 M HEPES sodium pH 7.5 2% *v*/*v* PEG 400 2 M ammonium sulfate

**Table 2:** Data collection statistics summary.

X-ray data collection statistics	
Beamline	BL44XU
Space group	P12_1_1
Temperature (K)	100
Wavelength (Å)	0.9
Resolution range (Å)	50.00–1.39 (1.41–1.39) *
Unit cell parameter	
*a*, *b*, *c* (Å)	38.99, 90.17, 40.60
α, β, γ (°)	90.00, 113.81, 90.00
Completeness (%)	99.5 (99.9) *
Mosaicity	0.35
Redundancy	7.3 (7.5) *
R_merge_	0.074 (0.612) *
Average I/σ (*I*)	33.358 (3.66) *
Unique reflections	385,687

* The value in parentheses are for the highest shell.
